# Primary mucinous cystadenocarcinoma of the spleen: a case report and literature review

**DOI:** 10.3389/fmed.2025.1662856

**Published:** 2025-10-17

**Authors:** Bo Zhou, Canyang Zhan, Sheng Yan

**Affiliations:** ^1^Department of Hepatobiliary and Pancreatic Surgery, The Second Affiliated Hospital, Zhejiang University School of Medicine, Hangzhou, China; ^2^Department of Neonatology, Children's Hospital, School of Medicine, Zhejiang University, Hangzhou, China

**Keywords:** primary mucinous cystadenocarcinoma, spleen, complete surgical removal, ectopic pancreas, case report

## Abstract

**Background:**

Tumors of the spleen are uncommon, and most represent metastases from primary organs. Primary mucinous cystadenocarcinoma of the spleen is an extremely rare tumor. Only 11 cases have been reported in the literature.

**Case presentation:**

Herein, we present a case of primary mucinous cystadenocarcinoma in the spleen in a 50-year-old woman. Abdominal CT and MRI revealed a 9.5 × 6.5 cm mixed solid-cystic lesion with heterogeneous enhancement of the mural nodules and septations, with secondary extension abutting the gastric fundus and pancreatic tail. Notably, tumor biomarker profiling demonstrated remarkable increases in CA19-9 (>12,000 U/ml) and CEA (41.5 ng/ml) levels. Following splenectomy, histopathology revealed the mass to be a mucinous cystadenocarcinoma. Given that metastatic cystadenocarcinoma is relatively common, investigations were performed to evaluate the primary site of malignancy. A whole-body PET-CT scan did not reveal any metabolically active lesions in any part of the body. Neither upper gastrointestinal endoscopy nor colonoscopy revealed any primary malignant lesions. Hence, it was reported as a primary mucinous cystadenocarcinoma of the spleen.

**Conclusion:**

The need for presenting this case is due to its rarity and because mucinous cystadenocarcinoma can be a rare differential diagnosis in cases of malignant splenic cysts.

## Introduction

Splenic neoplasms, whether primary or metastatic, constitute uncommon clinical entities. Histopathologically, primary splenic tumors are classified into haematolymphoid and non-haematolymphoid categories, with lymphoproliferative malignancies and angiosarcoma demonstrating predominance in the respective subgroups (1). Metastatic involvement of the spleen has been documented in 2.3%−7.1% of autopsy series from cancer patients, typically manifesting as terminal events in disseminated oncological disease (2, 3).

Within the spectrum of mucinous cystic malignancies, ovarian, appendix, and pancreatic origins account for more than 90% of reported cases (4). In contrast, primary splenic cystadenocarcinomas represent exceptionally rare clinicopathological phenomena. To date, 11 cases of mucinous cystadenocarcinoma of the spleen have been reported worldwide (5–15). Here, we report a case of primary mucinous cystadenocarcinoma of the spleen.

## Case presentation

A 50-year-old woman was incidentally found to have splenic cystic lesions measuring 7.2 cm in diameter in October 2016. The tumor marker expression levels were all normal. The patient subsequently underwent annual cross-sectional surveillance (ultrasound/CT), which revealed imaging characteristics consistent with a benign splenic cyst and dimensional stability over 8 years.

**Figure 1:**
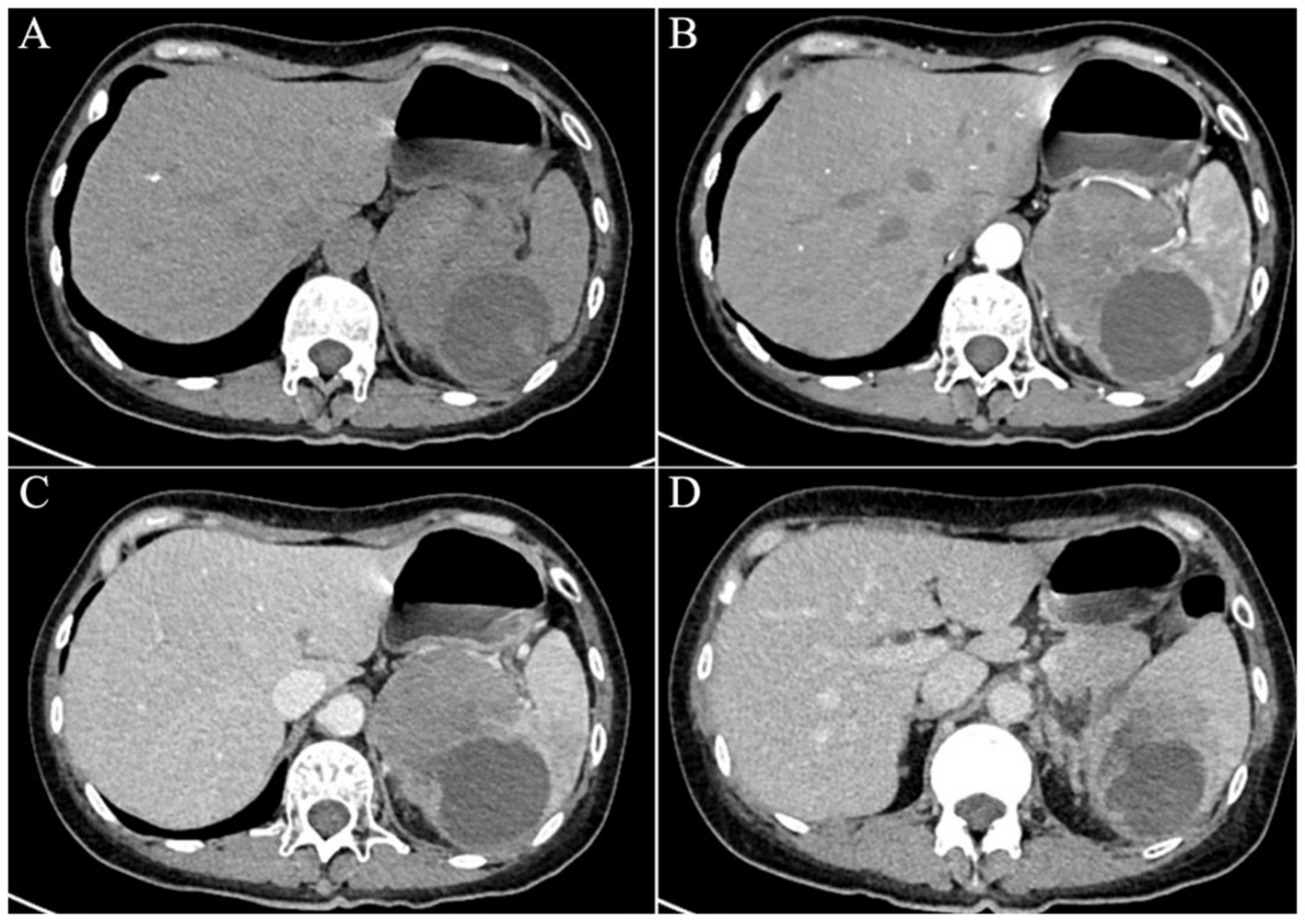
An abdominal computed tomography scan confirmed a 9.5 × 6.5 cm heterogeneous splenic mass with cystic degeneration **(A, B)**, demonstrating extrasplenic extension abutting the gastric fundus and pancreatic tail **(C, D)**.

**Figure 2:**
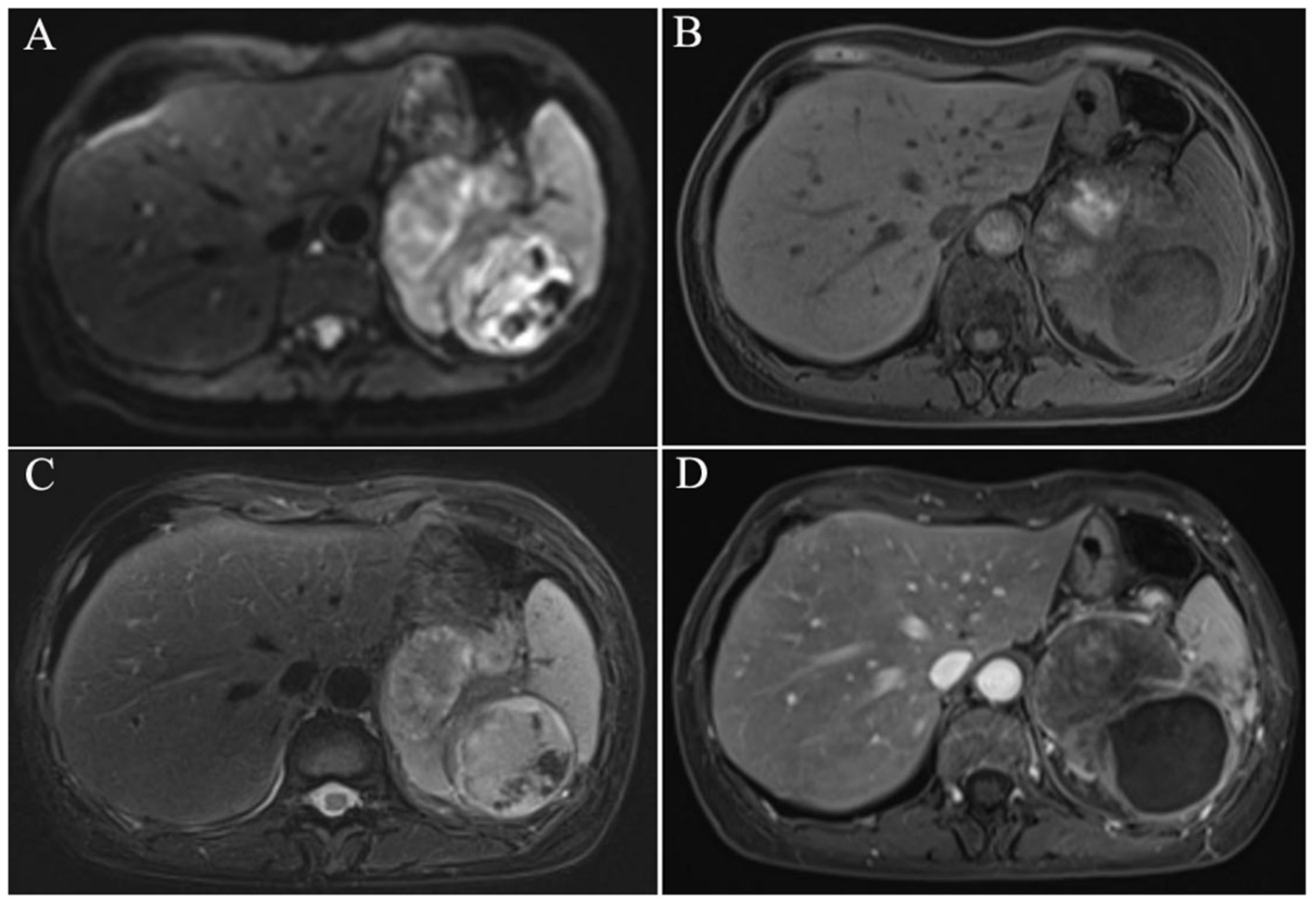
MRI revealed a mixed solid–cystic lesion with heterogeneous enhancement of the mural nodules and septations [**(A)** diffusion-weighted imaging, **(B)** T1-weighted imaging, **(C)** T2-weighted imaging, **(D)** Venous phase].

In November 2024, interval surveillance imaging revealed progressive enlargement of the splenic mass in the absence of abdominal pain, diarrhea, or fever. The woman had no history of lymphadenopathy, organomegaly, or signs of chronic liver disease. Contrast-enhanced abdominal CT revealed a 9.5 × 6.5 cm heterogeneous splenic mass with cystic degeneration, with secondary extension abutting the gastric fundus and pancreatic tail (Figure 1). Subsequent MRI corroborated these findings, revealing a mixed solid-cystic lesion with heterogeneous enhancement of the mural nodules and septations (Figure 2). Notably, tumor biomarker profiling demonstrated remarkable increases in CA19-9 (>12,000 U/ml), CEA (41.5 ng/ml), and CA242 (>1,000 U/ml) levels, whereas routine hematological, coagulation, and metabolic panels remained unremarkable.

The patient subsequently underwent laparoscopic splenectomy, where a mass was observed to apparently arise from the spleen abutting the tail of the pancreas and the greater curvature of the stomach but without obvious peritoneal disease. During surgical exploration, the tail of the pancreas was carefully dissected and separated intact without resection. Furthermore, wedge gastrectomy was also performed because the mass tightly adhered to the fundus of the stomach. The patient recovered well after surgery without any postoperative complications.

**Figure 3:**
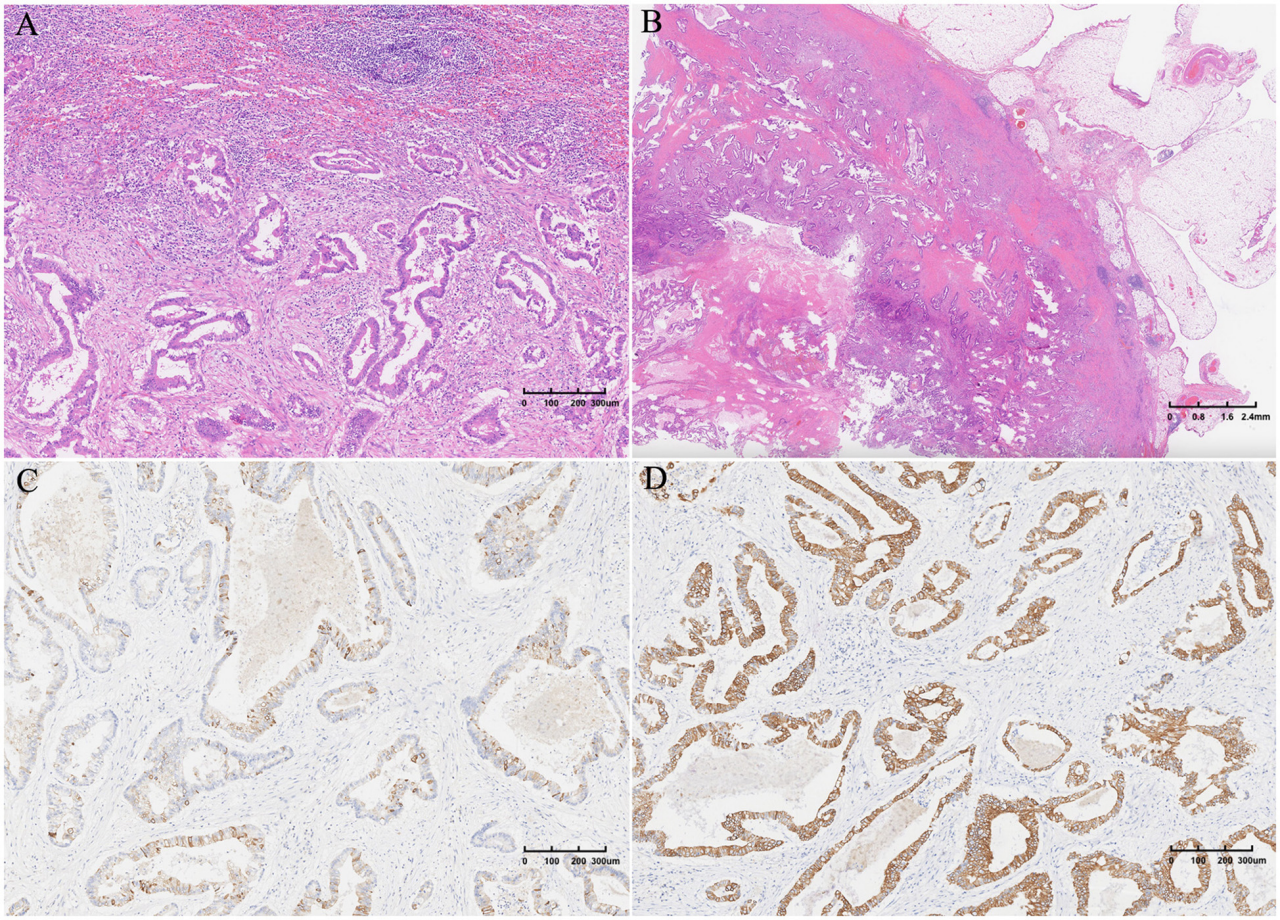
Postoperative histopathological examination revealed a spleen cyst covered by atypical irregular glandular epithelium invading the serosa of the gastric wall and the surrounding greater omentum, which contained mucinous fluid **(A, B)**. CK7 and CK20 antigen expression was detected via immunoenzymatic tests **(C, D)**.

Interestingly, dedicated sectioning and thorough microscopic examination of the entire specimen confirmed that the spleen cyst was lined by atypical irregular glandular epithelium, invading the serosa of the gastric wall and the surrounding greater omentum, which contained plenty of mucinous fluid (Figures 3A, B). Furthermore, the cyst lining was extensively sampled and shown to be composed of mucin-containing epithelial cells with architectural atypia. No ovarian stroma, pancreatic tissue, or lymph node metastasis were detected in the specimen microscopically. The Ki-67 proliferation index was 60%. Immunohistochemical staining was positive for CK7, CK20, and p53 (Figures 3C, D). The tumor was negative for WT1, TTF-1, ER, PR, PAX-8, and CDX-2 antigens. A middle-grade mucinous cystadenocarcinoma was diagnosed. The excision was complete (R0).

**Figure 4:**
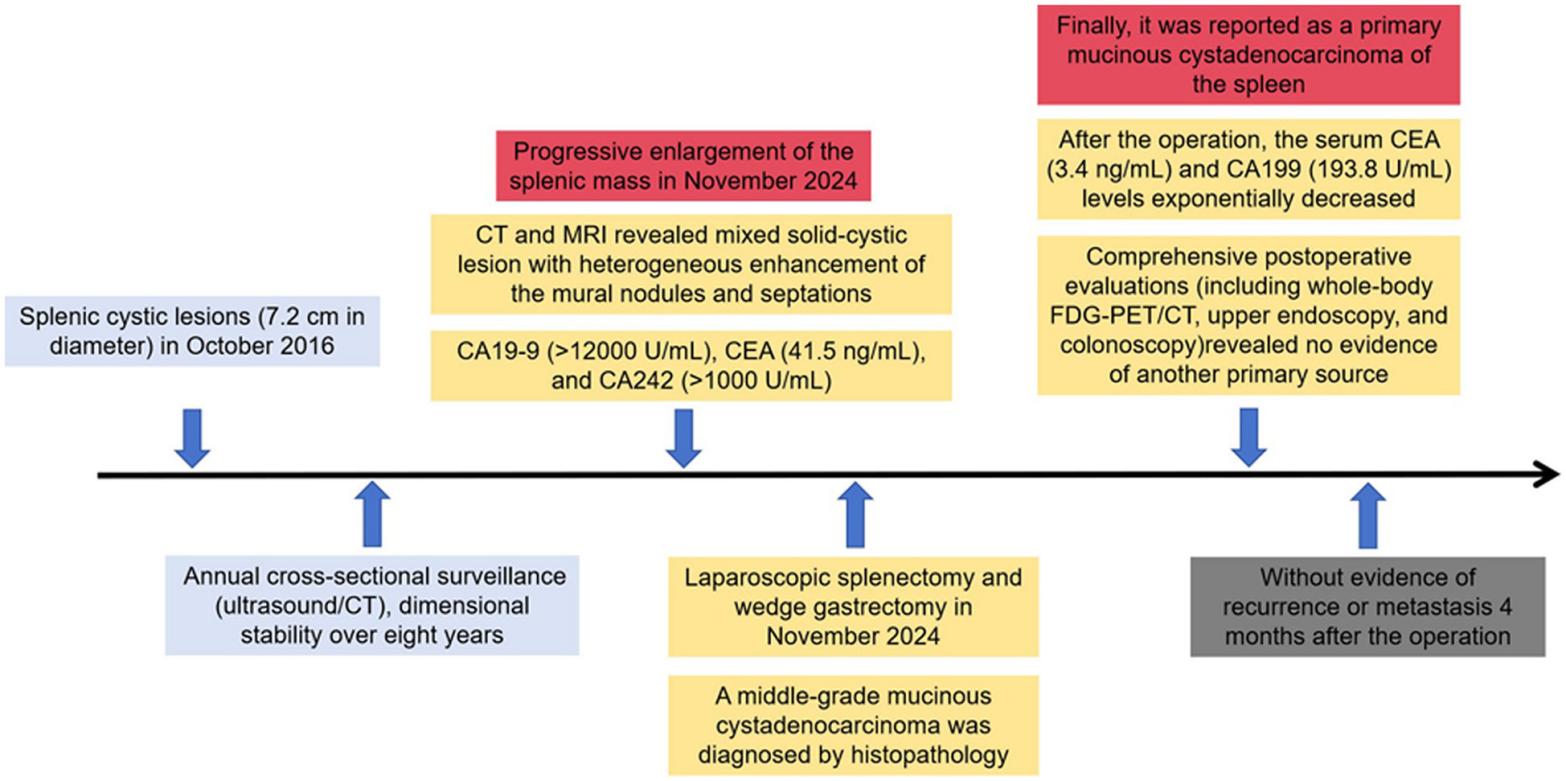
A timeline summarizing the main events of this case report.

After the operation, the serum CEA (3.4 ng/mL) and CA199 (193.8 U/mL) levels exponentially decreased. Owing to the nature of the tumor, the patient was also recommended to remain under the care of the ambulatory gynecology clinic and undergo PET examination in the postoperative period. Whole-body fluorodeoxyglucose (FDG)-PET/CT examination revealed no other primary foci of MCNs, especially in the ovaries. Upper gastrointestinal endoscopy and colonoscopy did not reveal any specific lesions. Hence, it was reported as a primary mucinous cystadenocarcinoma of the spleen (Figure 4). We offered the patient a treatment regimen similar to that used for mucinous cystadenocarcinoma of the gastrointestinal tract, with radical surgery followed by adjuvant chemotherapy. She has remained well without evidence of recurrence or metastasis 4 months after the operation.

## Discussion

Malignant tumors of the spleen can be classified as lymphoid, non-lymphoid or metastatic and most frequently originate from melanomas or breast or lung cancers (1). Primary involvement of the spleen in lymphomas is much rarer than splenic infiltration over the course of the disease, and the most frequently encountered infiltrates are non-Hodgkin's lymphomas originating from B cells (2, 3). Non-lymphoid malignant tumors are rare and include sarcomas, angiosarcomas and malignant teratomas.

While the ovaries, appendix, and pancreas account for the vast majority of primary mucinous cystadenocarcinomas, rare cases have been documented in other sites including the gastrointestinal tract and urachus. For the present case, these potential primary origins were systematically ruled out through comprehensive imaging and clinical assessment. In view of the clinical and pathological findings, the tumor in our patient was considered a primary splenic mucinous cystadenocarcinoma.

The pathogenesis of splenic cystadenocarcinoma remains unclear. Five hypotheses were considered: heterotopic pancreatic tissue; heterotopic intestinal tissue; invaginated mesothelium of the splenic capsule; local invasion from a pancreatic malignancy; and metastasis. Ectopic pancreatic tissue has 0.55–13.7% prevalence in autopsy series (16), although splenic localization represents merely 1% of these aberrant foci. While these embryological remnants typically remain clinically silent, their neoplastic potential mirrors that of orthotopic pancreatic tissue, with documented progression to adenocarcinoma (3). To conclude, that a tumor developed from the pancreas, the following conditions must be fulfilled: first, normal pancreatic tissue should be present in the organ together with a neoplastic component, and if possible, a transition should be histologically proven between them; second, a neoplastic lesion should not be found in the pancreas. Notably, our specimen exhibited no acinar/ductal differentiation or endocrine components despite detailed examination of multiple sections, excluding this etiology of heterotopic pancreatic tissue despite its predominance in historical reports (Table 1).

**Table 1:** Cases of primary mucinous splenic cystadenocarcinoma reported in the literature.

**Case**	**Authors**	**Sex/Age**	**Symptom**	**Size (cm)**	**Cysts (*N*)**	**Other features**	**Management**	**Proposed origin**
1	Shuman et al. (5)	F/42	Abdominal mass	9	2	NI	Splenectomy and distal pancreatectomy	Ectopic pancreas
2	Matsumoto et al. (6)	M/69	Abdominal pain, vomiting and constipation	4.7	Multi	Invasion into the splenic flexure of the colon	Splenectomy, distal pancreatectomy and left hemicolectomy	Pancreatic tail or ectopic pancreas
3	Hoshikawa et al. (7)	F/85	Diarrhea	19	Multi	NI	Splenectomy	Unknown
4	Morinaga et al. (8)	M/69	Abdominal mass	14.5	Multi	Elevation of serum CEA and CA19-9 levels	Splenectomy	Invaginated mesothelium of the splenic capsule
5	Zanetti et al. (9)	F/21	Abdominal pain	15	Multi	NI	Splenectomy and adjuvant chemotherapy	Ectopic pancreas
6	Hirota et al. (10)	F/68	Incidental finding	10	Multi	Ki-ras gene point mutation	Splenectomy and left nephrectomy, followed by delayed distal pancreatectomy	Ectopic pancreas
7	Nisar et al. (11)	F/69	Abdominal pain and breathlessness	20	Multi	NI	Splenectomy and adjuvant chemotherapy	Ectopic pancreas
8	Ohe et al. (12)	F/66	Abdominal pain	NI	Multi	Elevation of serum CEA and CA19-9 levels	Splenectomy and adjuvant chemotherapy	Invaginated mesothelium of the splenic capsule
9	Fujino et al. (13)	M/63	Abdominal distension	7.2	2	NI	Splenectomy and distal gastrectomy	Ectopic pancreas
10	Raul et al. (14)	M/61	Abdominal pain and distension	NI	Multi	NI	Splenectomy	Malignant transformation of an epidermoid cyst
11	Wlazlak et al. (15)	F/45	Incidental finding	18	1	NI	Splenectomy	Unknown
12	Our case	F/50	Incidental finding	9.5	1	Elevation of serum CEA and CA19-9 levels	Splenectomy, wedge gastrectomy and adjuvant chemotherapy	Unknown

Multi, multilocular, NI, no information.

Cysts of the spleen are divided into true (primary or epithelial-lined) and false (secondary or pseudocysts) cysts. Splenic mucinous cysts are cystic spaces that are lined by mucin-producing epithelium and that range from benign cystadenoma to malignant cystadenocarcinoma (17–20). Epidermoid variants demonstrate malignant potential through metaplastic-dysplastic progression to squamous cell carcinoma and mucinous cystadenocarcinoma (12). Immunohistochemical analysis revealed a CK7+/CK20+/p53+ phenotype with negativity for WT1, TTF-1, ER, and CDX-2. This profile is consistent with a mucinous cystadenocarcinoma and is discordant with origins in the lung, thyroid, endometrium, or ovaries. Although the phenotype shares features with gastrointestinal neoplasms, the possibility of a metastatic lesion was ruled out through a meticulous diagnostic workup. The absence of another primary source (pancreatic, gastrointestinal, and gynecological primaries) on contrast-enhanced CT/MRI, FDG-PET/CT, upper gastrointestinal endoscopy and colonoscopy conclusively confirmed the diagnosis of a primary splenic mucinous cystadenocarcinoma.

Radical en bloc splenectomy remains the therapeutic gold standard for multiple cystadenomas, large cysts and those infiltrating into splenic tissue, as cyst aspiration or partial resection risks peritoneal dissemination (17, 18). Intraoperative frozen section analysis is critical, given the histological overlap between benign and malignant cystic lesions. Intraperitoneal chemotherapy is required when there is an intraperitoneal spill of cystadenocarcinoma.

Advances in research continue to refine systemic treatment strategies for adenocarcinomas. Gastrointestinal adenocarcinomas, comprising esophageal, gastroesophageal junction, gastric, and colorectal subtypes, represent a leading cause of cancer-related mortality worldwide. It is primarily treated through a combination of surgical, chemotherapy, and radiation therapies (21). Progress in molecular profiling has enabled biomarker-directed therapies for specific patient subgroups, improving outcomes in populations such as those with HER2 amplification or Claudin18.2 overexpression (22, 23). The advent of immune checkpoint inhibitors (ICIs) has transformed the therapeutic landscape for gastrointestinal adenocarcinomas (24). However, predictive biomarkers beyond PD-L1 expression remain limited. Tumors exhibiting mismatch repair deficiency or high microsatellite instability (dMMR/MSI-H) are generally associated with a more favorable prognosis and derive substantial benefit from ICIs, despite some clinical heterogeneity. In advanced dMMR/MSI-H disease, ICIs demonstrate efficacy across treatment lines and are recommended as first-line therapy. In patients with non-metastatic dMMR/MSI-H cancer, increasing evidence suggests that perioperative and adjuvant chemotherapy may not provide benefit to the dMMR/MSI-H subgroup (24).

## Conclusion

In this report, we describe a rare case of primary mucinous splenic cystadenocarcinoma. Therapeutic success hinges on R0 resection combined with biomarker-guided adjuvant therapy, while long-term surveillance must address the undefined metastatic potential of this entity.

## Data Availability

The original contributions presented in the study are included in the article/supplementary material, further inquiries can be directed to the corresponding author.

## References

[1] Silver DS Pointer DT Jr Slakey DP. Solid tumors of the spleen: evaluation and management. J Am Coll Surg. (2017) 224:1104–11. 10.1016/j.jamcollsurg.2016.12.043 28126545

[2] Berge T. Splenic metastases. Frequencies and patterns. Acta Pathol Microbiol Scand A. (1974) 82:499–506. 10.1111/j.1699-0463.1974.tb00379.x 4854372

[3] Shi Z Zhao Q Lv B Qu X Han X Wang H. Identification of biomarkers complementary to homologous recombination deficiency for improving the clinical outcome of ovarian serous cystadenocarcinoma. Clin Transl Med. (2021) 11:e399. 10.1002/ctm2.399 34047476 PMC8131501

[4] Shiono S Suda K Nobukawa B Arakawa A Yamasaki S Sasahara N. Pancreatic, hepatic, splenic, and mesenteric mucinous cystic neoplasms (MCN) are lumped together as extra ovarian MCN. Pathol Int. (2006) 56:71–7. 10.1111/j.1440-1827.2006.01926.x 16445818

[5] Shuman RL Bouterie RL. Cystadenocarcinoma of the pancreas presenting as a splenic cyst. Surgery. (1976) 80:652–4. 982285

[6] Matsumoto M Kawamura Y Ishiguro M Nagai T. Cystadenocarcinoma of the pancreas manifested as a splenic cyst. Acta Pathol Jpn. (1981) 31:1089–96. 10.1111/j.1440-1827.1981.tb02020.x 7315312

[7] Hoshikawa T Hirata K Shiramatsu K Aizawa M Kimura H Hayasaka H. A case of mucigenic epitherial cyst in the spleen. J Jpn Clin Surg Soc. (1987) 48:421–4. 10.3919/ringe1963.48.421

[8] Morinaga S Ohyama R Koizumi J. Low-grade mucinous cystadenocarcinoma in the spleen. Am J Surg Pathol. (1992) 16:903–8. 10.1097/00000478-199209000-00009 1415909

[9] Zanetti G Riccioni L Gallo C Salfi N Martinelli GN. Splenic mucinous cystadenocarcinoma arising in heterotopic pancreatic tissue. Tumori. (1998) 84:606–10. 10.1177/030089169808400519 9862526

[10] Hirota M Hayashi N Tomioka T Murakami S Ohshima H Yamasaki K. Mucinous cystadenocarcinoma of the spleen presenting a point mutation of the Kirsten-ras oncogene at codon 12. Dig Dis Sci. (1999) 44:768–74. 10.1023/A:1026622111220 10219836

[11] Nisar PJ Zaitoun AM Lobo DN Rowlands BJ. Heterotopic pancreas in the spleen: malignant degeneration to mucinous cystadenocarcinoma. Eur J Gastroenterol Hepatol. (2002) 14:793–6. 10.1097/00042737-200207000-00015 12169992

[12] Ohe C Sakaida N Yanagimoto Y Toyokawa H Satoi S Kwon AH. A case of splenic low-grade mucinous cystadenocarcinoma resulting in pseudomyxoma peritonei. Med Mol Morphol. (2010) 43:235–40. 10.1007/s00795-010-0507-2 21267701

[13] Fujino Y Kanaji S Kawasaki K Tominaga M Kajimoto K. Mucinous cystadenoma of the ectopic pancreas with mucinous cystadenocarcinoma of the spleen in a male patient: a report of a case. JOP. (2015) 8:390–3.

[14] Raul SK Yadav B Sharma R Joseph M. Primary mucinous cystadenocarcinoma of spleen with pseudomyxoma peritonei: a very rare entity. Indian J Surg. (2020) 82:433–5. 10.1007/s12262-019-01978-1

[15] Wlazlak M Grzasiak O Wierzchniewska-Ławska A Hogendorf P Durczyński A Strzelczyk J. Mucinous cystadenocarcinoma of the spleen - a very rare case of a primary splenic MCN. Pol Przegl Chir. (2020) 93:1–5. 10.5604/01.3001.0014.5754 34057428

[16] Dolan RV ReMine WH Dockerty MB. The fate of heterotopic pancreatic tissue. A study of 212 cases. Arch Surg. (1974) 109:762–5. 10.1001/archsurg.1974.01360060032010 4420439

[17] Gupta J Gupta A. Ruptured primary mucinous cystadenoma of spleen leading to mucinous ascites. BMJ Case Rep. (2019) 12:e231212. 10.1136/bcr-2019-231212 31753825 PMC6887401

[18] Reta BK Beker AM Hagos HH Weldegebriel MH Kidanu GT Zeray MA. Rare case of primary mucinous cystadenoma of spleen: a case report. Int J Surg Case Rep. (2025) 126:110782. 10.1016/j.ijscr.2024.110782 39729897 PMC11741019

[19] Singh O Gupta S Shukla S Mathur RK. A rare case of primary mucinous cystadenoma of spleen. J Clin Med Res. (2009) 1:237–9. 10.4021/jocmr2009.09.1257 22461876 PMC3299188

[20] Lawgaly SA Eldruki S. A rare case of mucinous cystadenoma of the spleen in Libya. Qatar Med J. (2021) 2020:41. 10.5339/qmj.2020.41 33447540 PMC7784710

[21] Brandi G Ricci AD Rizzo A Zanfi C Tavolari S Palloni A. Is post-transplant chemotherapy feasible in liver transplantation for colorectal cancer liver metastases? Cancer Commun. (2020) 40:461–4. 10.1002/cac2.12072 32762027 PMC7494063

[22] Ricci AD Rizzo A Brandi G. DNA damage response alterations in gastric cancer: knocking down a new wall. Future Oncol. (2021) 17:865–8. 10.2217/fon-2020-0989 33508962

[23] Klempner SJ Janjigian YY Wainberg ZA. Claudin18.who? Examining biomarker overlap and outcomes in claudin18.2-positive gastroesophageal adenocarcinomas. ESMO Open. (2023) 8:100778. 10.1016/j.esmoop.2022.100778 36791669 PMC9958250

[24] Strickland MR Lander EM Gibson MK Ilson DH Ajani JA Klempner SJ. Gastroesophageal adenocarcinomas with defective mismatch repair: current knowledge and clinical management. J Natl Compr Canc Netw. (2024) 22:e237103. 10.6004/jnccn.2023.7103 38503041

